# Diving into the Resolution Process: Parent’s Reactions to Child’s Diagnosis

**DOI:** 10.3390/ijerph20043295

**Published:** 2023-02-13

**Authors:** Yael Barak-Levy, Bilha Paryente

**Affiliations:** Department of Early Childhood Education, Achva Academic College, Arugot 7980400, Israel

**Keywords:** parents of children with special needs, counseling parents, categorical methods, qualitative methods, reaction to diagnosis

## Abstract

This research presents an in-depth observation of parental resolution regarding a child’s diagnosis with special needs to help counsellors understand the complexities of parental coping. Sixty-two parents of children with Autism Spectrum Disorder/Intellectual Developmental Delay participated in a Reaction to the Diagnosis Interview and a semi-structured interview. Categorical analysis revealed that 59.7% of the parents had reached resolution, with approximately 40% emotional orientation, 40% cognitive orientation, and 20% proactive orientation. Content analysis revealed three themes: emotions—feelings of guilt, shame, and emotional breakdown; thoughts—fear of stigma and concern for the child’s future; actions—concealment, seeking support, and attempts to reject the results of the diagnosis. Whereas most parents were diagnosed as having reached resolution, the content analysis still found complex subject matter suggesting lack of resolution. Research findings show that counsellors should identify the intricate emotional dynamics of parents coping while being cautious of premature coping categorization.

## 1. Introduction

The arrival of a new child into the family is usually received with feelings of elation. However, when a child is diagnosed with disabilities, all the expectations and hopes parents had for a healthy child cease [1,2,3,4]. Parents bear the main responsibility for their children’s social, emotional, and academic [5]. As parents, every pain or problem our child feels affects us deeply [6]. Therefore, the realisation that a child’s future accomplishments and achievements may be hindered due to the child’s diagnosis with special needs can be very worrisome and stressful for parents [7,8]. These parents must meet the typical parental demands along with the tasks and responsibilities deriving from the child’s condition, all of which have been found to impact parental well-being [9], parent–child interactions, and familial relationships [10]. Parents of children with disabilities have been found to be prone to feelings of shame, guilt, sadness [11], anger, and disappointment [8]. They have been found to display ongoing symptoms of depression and psychological difficulties [7,11] and to portray a distorted view of their child’s abilities (e.g., [12].

The reaction of such parents to their child’s diagnosis has been widely debated in theoretical literature. Over time, researchers established the concept of ‘resolution’ with the child’s diagnosis as a process of acceptance, the end of active grieving, and a refocus on present and future realities [3,9]. Parents who were found to be unresolved, on the other hand, were shown to engage in ongoing bereavement [4]. These parents have been found to be more depressed and stressed than parents that have reached resolution [11,13].

In order to recognize individual differences in coping styles, Pianta and Marvin [14] developed a reliable, semi-structured interview tool—the Reaction to the Diagnosis Interview (RDI). The RDI consists of three subcategories of a resolved coping style (action, feeling, and thinking) and six subcategories of an unresolved coping style (emotionally overwhelmed, angry, neutralizing, depressed, cognitive distortion, and disorganization). Whereas this analysis created an important foundation for understanding parents’ coping, it is characterized as a categorical classification. 

Previous research has revealed diverse rates of resolution among parents of children with various disabilities, ranging between 18% and 72% (e.g., [6,9,15,16]. Quite a few studies have examined parental reactions to the diagnosis of children with Autism Spectrum Disorder (ASD) (e.g., [9,17,18] and of children with Intellectual Developmental Delay (IDD) [6,19]. Varied resolution rates have also been found in certain qualitative studies [4,17]. Even so, these rates pointed to a fair number of parents who were resolved, well-adapted, and functioning effectively. However, at the same time, they have shown that quite a few parents have difficulty achieving resolution [6,20,21]. These findings are especially important as implementations of the RDI found that parents classified as resolved supported their child’s functioning and displayed a better quality of parenting, secure child–parent attachment, a productive social life, and so on [3,4,6,16]. In turn, parents classified as unresolved exhibited inadequate coping strategies, poorer health, passive and angry social interactions, false beliefs about the child’s diagnosis and a state that may lead to insufficient or inappropriate parental decision-making (e.g., [3,4,16], higher parenting stress, and children’s insecure attachment [16].

It is important to point out that when defining the resolved coping styles, Marvin and Pianta [22] highlighted the fact that parents who were found to be resolved showed a shift from feelings of devastation to coping strategies, but still acknowledged the appearance of negative feelings and thoughts from time to time. Thus, while a sense of resolution is associated with improved parental mental and physical states [16,18,22] and a positive and encouraging style of interaction with the child [9,16], raising a child with disabilities remains a complex and difficult journey [23]. 

**Figure 1:**
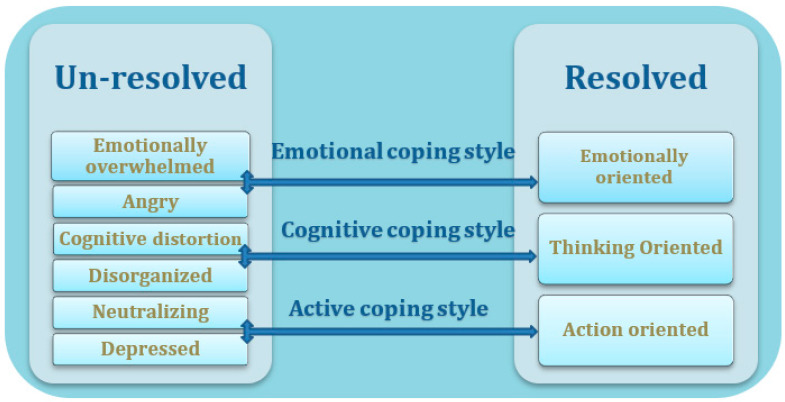
A visual display of the continuum approach to RDI categories.

A previous study [6] suggested viewing the resolution process as a continuum. This view assumed that a parent’s style of emotional response would be evident in both positive and negative sub-categories of resolution. The subcategories of the RDI were viewed as three distinct coping styles: The *emotional style* included the resolved emotional orientation and the unresolved reactions of being emotionally overwhelmed and angry. The *active style* included the resolved action orientation and the unresolved depressed/passive reaction. Lastly, the *cognitive style* included the resolved thinking orientation and the unresolved neutralizing, disorganized/confused, and cognitive distortion reactions. This theoretical view allowed for a more holistic, in-depth understanding of parents’ coping styles and emotional reactions to their child’s diagnosis. A recent review article [16], which mapped 47 research papers regarding parents’ reactions to their child’s diagnosis, also recommended viewing resolution as a continuous phenomenon. Therefore, this continuum approach [6] was chosen as an outline for the current study (see Figure 1). 

Viewing parents’ coping manner as a continuum and aligned with an underlying personality style [8] can be helpful for education, health, and emotional therapy professionals as they try to assist parents on their journey toward resolution and acceptance regarding their child’s diagnosis and practicing healthy and content parenting, attunement to their child’s needs, and overall better family functioning [24]. This well-rounded view on the emotional process the parents undergo may deepen the guidance that parents can receive from the assistance offered to them by the health and education systems. 

Yet, even with the continuum approach, the RDI results are categorical, as they assign a parent to a certain coping style. The use of a qualitative methodology can, therefore, be helpful in broadening this categorical view. Moreover, it can enable an in-depth perspective that promotes more precise understanding of the dynamics of the processes the parents undergo. This understanding can help at two levels: the theoretical level expands the contents behind the categorical classifications, and the practical level enables counsellors to improve the precision of the help they can offer parents during and following diagnosis. 

It is, therefore, clear why several previous qualitative studies have chosen to explore the coping styles of parents of children with disabilities (e.g., [25,26,27,28]. To widen the understanding of these intricacies, this study supported the categorical data with a thorough qualitative content analysis of an in-depth interview with the parents. The interview was structured so as to fully understand the parents’ emotional context, actions, and thought processes and allowed us to see simultaneously the categorical classifications as well as their underlying contents. 

The purpose of the current study was to take a deeper look into the cognitive, emotional, and practical processes parents undergo as they receive their child’s diagnosis as having special needs. This insight can help educational staff and counsellors to understand the complexities of parents’ emotions, thoughts, and behaviours, as well as whether they were diagnosed as resolved or unresolved. Stressing the intricacies of the resolution process is vital in order to offer more tailored and precise insights to counsellors accompanying parents on their journey toward resolution with their child’s diagnosis. 

In order to reach such insights, our first two research questions examined our categorical data, while the third question expanded that information with an in-depth qualitative content analysis:

1. What was the prevalence of resolution with the diagnosis among parents of children diagnosed with special needs during the last two years?
2. What was the distribution of the parental coping styles in regard to the continuum?
3. What contents characterize the different coping styles of parents of children with special needs?

## 2. Methods

This study was designed as a mixed-methods research (MMR), combining both categorical and qualitative methods [29]. One of the advantages of combining different research methods is to draw more effective and accurate conclusions using the results of one method to clarify the results of the other. In this case, the analysis of the interviews helped to explain the categorical results. 

### 2.1. Sample

#### 2.1.1. Participants

The research sample consisted of 62 parents (42 mothers and 20 fathers, each from a different family) between the ages of 28 and 45 (*M* = 37.1; *SD* = 5.05). They had been married for between 5 and 23 years (*M* = 14; *SD* = 1.1). All the children in question were between 4 and 5 years old (*M* = 4.5; *SD* = 1.22), and their birth order ranged from first to fourth (*M* = 2.2; *SD* = 1.04). All the children in question had been diagnosed with ASD and/or IDD by developmental neurologists. After being diagnosed, they were placed in a special education kindergarten in the centre or south of Israel.

Children with IDD either have IQ scores that are, at least, two standard deviations below the mean, or demonstrate impairments in at least two of the following adaptive functions: receptive language, expressive language, cognitive/visual reception, fine or gross motor skills, or adaptive behaviour [30]. ASD is defined as a permanent and constant deficiency in social communication and interpersonal interactions. This deficiency can be seen in poor socioemotional reciprocity, e.g., difficulty in conversing; limited emotional sharing; inability to initiate social interactions or respond to others’ social initiatives; poor nonverbal communication in social interactions; deficiency in developing and maintaining age-appropriate emotional relationships; limited and repetitive interests, activities and behaviours; hyper- or hyposensitivity to sensory stimuli [31,32,33].

#### 2.1.2. Sampling Method

As mentioned, the sample consisted of parents of children who had been placed in special education kindergartens in the centre and south of Israel were chosen. Two kinds of kindergartens were approached: kindergartens for children with medium to low function ASD and kindergartens for medium to low function IDD. After receiving approval from the supervisors and kindergarten teachers, we asked parents to participate. We interviewed about 30% of the parents of each kindergarten in accordance with their willingness to participate in the study. In order to focus on the initial reactions of parents as they cope with their child’s diagnosis with special needs and placed in the special education system, only parents of children between the ages of 4 and 5, who had been diagnosed within the last 2 years, were recruited. To ensure a homogenic sample, parents with two children with special needs were excluded, as were single parents, as this may act as a stress factor and alter their coping strategies.

### 2.2. Measures

#### 2.2.1. Categorical Measure

Parental resolution with the child’s diagnosis was assessed using the Reaction to Diagnosis Interview (RDI) [14]. Each parent also participated in an audio-recorded semi-structured interview. Parents were asked to: 1. recall the period when they began noticing something was wrong with their child’s development; 2. explain how they felt at that time and whether there were changes to those feelings; 3. describe the events and emotions surrounding the time they received the diagnosis; 4. explain how their feelings have changed since the time of the diagnosis; 5. detail whether they have been searching for existential or other reasons for their experiences. Coding of the recorded interviews was conducted by two trained coders using the RDI manual in a holistic coding approach (see [14]). 

#### 2.2.2. Qualitative Measure

The semi-structured interview was decided on as the tool of choice. According to Shkedi [34,35], the semi-structured interview is the most suitable method for documenting the narrative of participants’ meaningful experiences.

All interviews lasted between 60 and 90 min and were conducted by research assistants trained by the researchers. Interviews were performed in random order; they were audio recorded and then transcribed. The interviews were guided by five predefined topics, beginning with questions about participants’ parenting in general. These five predefined topics were as follows:

1. Please describe the moment you were informed of your child’s diagnosis.
2. Describe your parenting considering the diagnosis.
3. Describe how do you cope on a daily basis.
4. Describe your emotions while coping with day-to-day situations.
5. Describe how do you perceive your parental role towards your child with special needs?

Finally, parents were asked if they would like to provide additional details regarding parenting a child attending a special educational kindergarten.

### 2.3. Data Analysis

As mentioned, data obtained from the RDI were coded into categories according to the RDI manual [14] by trained coders. In accordance with our first two research questions, the categorical analysis gave us a clear view of the prevalence and distribution of parents’ coping styles. A parent was classified as resolved when elements of resolution were predominant (e.g., a sense of change since diagnosis and assertion of moving on in life, suspending the search for a reason and a realistic representation of a child’s abilities). Parents diagnosed as resolved can be sub-classified as feeling-, thinking- or action-oriented. 

A classification of unresolved was chosen if elements of lack of resolution were more predominant. These include cognitive distortions, ongoing search for reasons, focusing on the past, detachment from feelings, and an incoherent narrative. Parents classified as unresolved can be categorized as emotionally overwhelmed, angrily preoccupied, neutralizing, depressed/passive, having cognitive distortion, and disorganized/confused.

Two trained coders rated all interviews and reliability was reached, coding 20% of the sample. Judges had an inter-coder agreement of 100% on the resolved/unresolved classification and 88% on the subcategories. The reliability between judges was kappa = 0.85, *p* ≤ 0.001, which is considered an outstanding measure of agreement [36]. All disagreements were discussed and resolved by consensus.

Content analysis was conducted using the data obtained from the interviews. Transcripts were read and reread several times from start to finish by the researcher and by another content analysis expert, and only afterward were the focused analysis conducted and relevant quotes selected. The content analysis was conducted according to the guidelines and stages outlined by Shkedi [34,35], as a transparent and systematic method [37]. In the first stage—*open coding*—data are gathered and categorized according to themes, i.e., major topics that emerge repeatedly, often corresponding to the interview questions. In the second stage—*mapping*—new conceptual parameters are used to sort the data in categories. Throughout the analytic stages, the researcher identifies, compares, and discusses the findings with a colleague. In the third stage—*selective coding*—categories and themes are arranged hierarchically as a tree and then organized according to the development of the narrative. In the current study, the researcher and the additional expert were both professional female therapists, psychologists, and mothers, both born in Israel and thus native speakers of Hebrew. They analysed the data separately at each stage, and interrater reliability was then achieved by comparing their findings (90%), thus contributing to the trustworthiness of their interpretations.

### 2.4. Ethical Considerations

The researchers promised to maintain participants’ anonymity (names mentioned here are pseudonyms). Shlasky and Alpert [38] claimed that any written record or observation of phenomena is consciously or subconsciously tainted with the observer’s ethical, political, and moral attitudes. By definition, qualitative research engages in an attempt to reconstruct the reality observed; in this study, every effort was made to convey the voices of the participants as faithfully as possible. Furthermore, the research was approved by the Institutional Ethics Board of the academic institution in which the authors are employed, and all participants signed consent forms (Approval code: 0071).

### 2.5. Research Procedure

Data collection took place between November 2019 and February 2020. All interviews were conducted in the participants’ area of residence. The precise location took into consideration the participants’ convenience and the degree of privacy needed to conduct the interview effectively. All interviews were conducted face-to-face with only the interviewer present, and each lasted approximately 60–90 min. The participants gave their consent to have the interviews recorded and were assured that anonymity would be maintained. Each interview began by positing the following request: “This study is about the parenting experiences of a child who was diagnosed and placed in a special educational kindergarten. I would appreciate it if you could share with me some of your parenting experiences.” At the end of each interview, the researcher thanked the interviewee. Following the verbatim transcription of the recorded interviews, the researcher and the second expert listened to the recordings while reading the corresponding transcripts to identify possible omissions or transcription errors.

## 3. Results

### 3.1. Categorical Results

To answer our first research question, examining the pervasiveness of resolution within parents of children with disabilities, the proportion of parents classified as resolved and unresolved was calculated (see Table 1). The results showed that 59.7% of parents were classified as resolved and 40.3% as unresolved. Regarding the parents’ gender, 55% of the mothers were classified as resolved and 45% as unresolved. In addition, 68.2% of fathers were classified as resolved and 31.8% as unresolved. When focusing on the child’s gender, 47.4% of parents of girls were classified as resolved, whereas 65.1% of parents of boys were classified as resolved.

Next, proportions of all subcategories of both ‘resolved’ and ‘unresolved’ were calculated to examine the second research question. Within the parents classified as resolved, approximately 23% of parents exhibited an emotional orientation, with the same frequency shown for the thinking orientation. Approximately 14.5% displayed an active orientation. The analysis of the parents classified as unresolved revealed that most were emotionally overwhelmed (~14.5%); almost as many exhibited cognitive distortion (~9.7%); and a few were depressed/passive (4.8%), disorganized/confused (4.8%), angry (3.23%), and neutralizing (3.23%).

To answer the second research question, proportions were calculated for the three distinct coping styles in accordance with the continuum framework (see Table 2). The vast majority of parents were equally divided between the emotional (40.3%) and cognitive (40.3%) coping styles. Only 19.4% of parents displayed an active style of coping with their child’s diagnosis.

### 3.2. Qualitative Results

**Figure 2:**
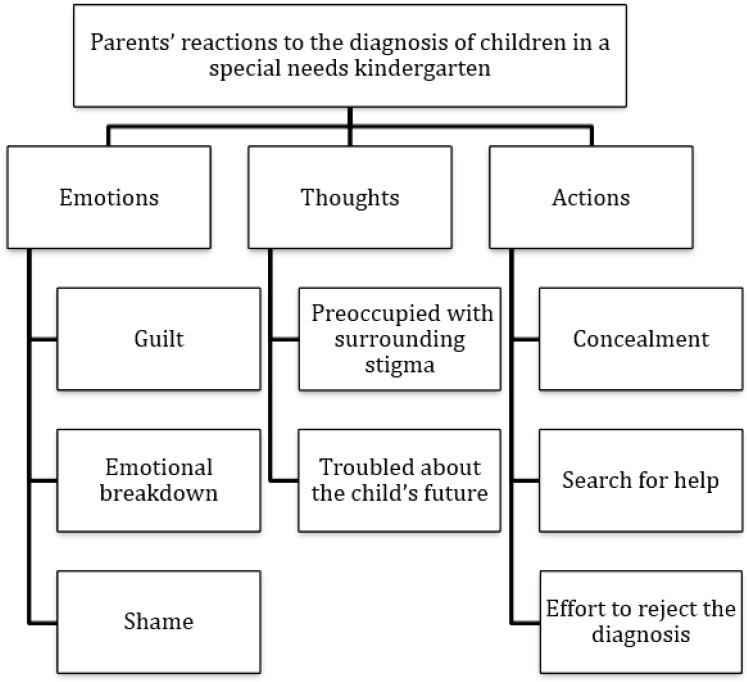
Map of themes as derived from data analysis.

Following the third research question, and in order to allow for an in-depth understanding of the parents’ coping styles, content analysis was conducted using the data obtained from the interviews. In light of the theoretical outline that views the RDI categories as a continuum, this investigation described the content expansion of the emotions, thoughts, and actions of parents as they dealt with their child’s diagnosis (see Figure 2). 

### 3.3. Emotions

“From the moment we received the diagnosis, I felt that my world was in ruins and that I was trapped in a profoundly intense storm of emotions. I felt a combination of many unbearable things to the point of collapse. I felt as if I was on a tempestuous sea, being beaten by the waves.”

This image, given by a mother when asked to describe her reaction to her child’s diagnosis, reflects the emotional maelstrom many parents find themselves in upon hearing their child’s diagnosis.

An overview of the emotions raised by the parents in the interviews show they were flooded with various emotions, the three main ones being guilt, emotional breakdown, and shame.

#### 3.3.1. Guilt

Fifty-two parents shared feelings of guilt that had overwhelmed them on receiving the diagnosis of their child. Guilt (mostly among mothers) focused on the idea that the parent had not been good enough and maybe that was the reason something had gone wrong with the child. For example, Dana (f., 32) said: “There were moments when I would just blame myself, that I hadn’t been good enough toward him. I felt guilt. I felt that I wasn’t doing enough for him.”

Rivka (f., 33) blamed herself because her daughter’s disability might have been less significant if she had given her more attention, “because I could have given her more attention when she was a baby and maybe her delay would have been less drastic.” The guilt also revolved around the fact that the parent had missed the need for a diagnosis and had not seen the problem before the diagnosis findings. For instance, Dvora (f., 37) stated: “I don’t understand how I didn’t rely on my intuition as a mother from the beginning, when I felt that something was not right with Omer... I feel a bit guilty.”

In addition, there was also guilt about the genetic inheritance the parents had passed on to the child. Yehoshua (m., 44) shared those feelings with us:

“Sometimes I look at his facial features and I see myself when I was young. Especially when comparing pictures, putting one next to the other. My stomach turns over and I feel that I am responsible; those are my genes.”

The feelings of guilt the parents mentioned related mainly to the sense that perhaps they could have done more for their child, that perhaps if they had made more effort, been more available, paid more attention or even had a different genome, this would not have happened, and their child’s diagnosis would have been different. These emotions seemed to be hard to bear, and parents mentioned that they cope with them on a daily basis. 

#### 3.3.2. Emotional Breakdown

Thirty-eight parents stated that they had reached a state of emotional breakdown in dealing with their child and with the diagnosis on a daily basis. All of them also indicated the physical aspect that accompanied the emotional side. Yarona (f., 39) shared her feelings: “It led the situation to a bad place, causing pressure in my chest, a kind of heart attack. After a week in bed, constantly crying about the situation and about Daniel and three tranquilizers…”

Parents spoke of breakdowns accompanied by physical pain upon hearing their child’s diagnosis. They felt their strength had ebbed away, that they felt physically ill, and they just wanted to rest. There was a combination here of emotional fatigue and physical pain.

#### 3.3.3. Shame

Forty-five of the parents mentioned their feelings of shame stemming from their child’s diagnosis. For example, Yosef (m., 29) told us:

“I was ashamed in the beginning about what the neighbors or our friends would say about the fact that I had a child like that. It took me time to take him out to parks and playgrounds because it’s really hard. He used to sprawl out on the ground in the middle of the park.”

Parents were also ashamed about their child being placed in a special education kindergarten. For example, Alma (f., 28) told us:

“Of course, I had concerns about what they would say about my daughter being placed in special education. How would the surroundings react? I was afraid it would be embarrassing…My husband, of course, found it more difficult than I did.”

David (m., 35) said: “It’s clear that I was worried. You worry about what they will say about my son being in a special kindergarten … It’s a little like being kind of embarrassed.…”

The parents shared with us their feelings of shame, which included the shame of the child’s inappropriate behaviour in public and of the child’s placement in a special education kindergarten. The parents experienced such shame that it made them want to conceal information about their child.

### 3.4. Thoughts

The parents were preoccupied with two main thoughts: worry about the stigma that their immediate environment would place on the child and about their child’s future.

#### 3.4.1. Preoccupation with Surrounding Stigma

Thirty-five parents raised their fears of the stigma and its effect on their children. This fear stems from the placement of their children in a special education kindergarten and the fact that people will find out about the diagnosis. Shani (f., 35) was bothered by the stigma people would attach to her child following his diagnosis: “I was so afraid that the surroundings would know about the diagnosis and start stigmatizing Omri as autistic and detached.”

Michael (m., 43) related that his fear of his son’s being labelled led him to leave his son in a regular kindergarten class: “The special education system sounded to me like a nightmare. I didn’t want my son to bear the label of a special education child.” Omer (m., 45) spoke of his difficulty in the transition because of his fear of stigma: “The transition was really hard for me. I was fearful and I thought about how she would feel there and what people would think of her and that they would label her.” 

The parents were bothered by the stigma that would be attached to their child following the diagnosis and the fact that the child was in a special education kindergarten. They feared that the stigma would irreversibly accompany them and their child to the end of their days. 

#### 3.4.2. Troubled about the Child’s Future

Fifty-eight parents expressed their concern for the future of their child. They voiced many fears about the possibility that their children would not be independent and would not be able to take care of themselves. For example, Yitzchak (m., 40) mentioned his concern about how his child would manage in the future without him: “Yes. It’s frightening. What will happen if he is not independent? If he needs us until the end of our lives and we won’t be able to help him?” In addition, concern was also raised about the ability of the child to find a partner and have their own family life. Karin (f., 38) stated: “You don’t know what will happen in the future, how she will grow up, how she will communicate, whether she will find a life partner, whether she will get married.”

The parents were concerned that their child would not be able to manage in the future independently, that they would not be able to find a partner, manage a relationship, and build their own nuclear family. 

### 3.5. Actions 

The parents described their coping in terms of behaviour in three ways: concealment, seeking help and support, and attempting to reject the diagnosis.

#### 3.5.1. Concealment

Forty of the parents brought up the issue of how they dealt with concealment. Here, concealment relates to hiding the actual diagnosis of their child. Irit (f., 35) said that she actually avoided leaving her home:

“We saw our friends and family less. We avoided leaving home with him. We didn’t speak freely with our close surroundings. We were always busy hiding, in the family, that perhaps we would succeed in narrowing the gap as time passed.”

In addition, parents also spoke of their desire to conceal the fact that their child had been placed in a special education kindergarten. Uri (m., 38) said: “At the beginning, we didn’t tell anyone about the diagnosis and the recommendation for a special education kindergarten. We didn’t want anyone to know.”

As a result of the feelings of shame and of the fear of stigma, parents shared that they made efforts to conceal the diagnosis and the placement of their child in a special education kindergarten. The concealment included not talking about matters and often even a decision not to leave the house so that people would not see the issues in public.

#### 3.5.2. Search for Help and Support

Thirty-five of the parents spoke of their desire to obtain help from their families. Sigalit (f., 34) recalled:

“I was surprised at how much the family could help and give support. Even now that my Hodaya is six years old, they still support and help. They actually read about all kinds of treatments and call to tell me. In general, I can say that without my family, managing would be much more difficult. Thank God that we have one another.”

It is interesting that with all the efforts of concealment, the parents also spoke about seeking help—both the support of getting advice on recommended treatments and technical help, mainly from family members.

#### 3.5.3. Effort to Reject the Diagnosis

Forty-five of the parents spoke of their hopes of rejecting the diagnosis that they had received for their children. This hope was based on the idea that if they could obtain another opinion, the diagnosis would be different. For instance, Meir (m., 42) stated:

“I was in shock. I felt as though my world was being destroyed… I didn’t understand what it meant… What is autism…I was in denial; I said that it couldn’t be happening to me…Maybe it was a mistake. Maybe if I make an effort and go for another opinion with another doctor, they will tell me it was a mistake…”

In addition, hopes were also based upon the fact that things would improve as time went by, and the diagnosis would become invalid. Yaira (f., 45) explained: “What was limited intelligence? That couldn’t be. I thought that would change as time went by. He would grow and improve, and he would be like everyone else…”

The effort to reject the diagnosis revolved around the hope that there had been a mistake and perhaps going for a second opinion would uncover it. In addition, there were also efforts to work with the child to improve their condition and change the diagnosis—a kind of parental fantasy of a “happy ending.”

## 4. Discussion

The current study examined the processes of resolution and coping styles of parents who, in the previous two years, were told that their child had been diagnosed as having Autism and/or Intellectual Developmental Delay. This was done to help counsellors optimize their accompaniment of parents through their process of resolution. The uniqueness of this study is its in-depth look at the contents underlying the parental paths of coping with their child’s diagnosis by combining the methods of categorical research with qualitative research.

The first stage examined the categorical results emerging from the RDI. The findings showed that close to 60% of the parents in the study had reached resolution with the diagnosis of their child, despite the short time that had passed since receiving it.

These findings concur with previous studies that have examined the parameters of resolution for parents of children with special needs and found that resolution rates range from 18% to 72% (e.g., [6,9,15,16]. However, taking into consideration that we are talking about parents of kindergarten children who had had a relatively short time to undergo the resolution process, it is surprising to see that the percentage of parents who have reached resolution actually matches the top end of the bar for earlier studies.

The categorical analysis further presents the proportional distribution of parental coping styles. Some 40% of the parents adopted the emotional coping style, and a similar percentage adopted the cognitive parenting style. Interestingly, less than 20% adopted an active coping style. As mentioned, a recent review [16] recommended taking a continuous approach to resolution, yet only one study to date presented resolution data as coping continua [6]. This study also found that the cognitive and emotional coping styles are more frequent than the active coping style. 

The findings of the high percentage of parental resolution such a short time after the diagnosis and the coping styles that are more focused on emotions and thoughts than on action might be an indication of parents who have undergone more intensive processing. Previous research showed that parental emotional availability corresponds with higher levels of resolution [13]. Perhaps this process is bidirectional, and parents who allow themselves to delve into mental and emotional processing and do not focus mainly on actions reach their resolution with the diagnosis of their child in greater numbers and in a relatively short process.

Through the structuring of coping continua according to parental emotions, thoughts, and actions, a qualitative content analysis of the semi-structured interviews was performed. The theme of emotions showed that for most parents, the most overwhelming emotions were guilt, shame, and emotional breakdown. For the theme of thoughts, most parents were troubled by the stigma imposed by their surroundings and concerns about the child’s future. For the theme of actions, most parents spoke of concealment, seeking help and support, and an attempt to alter the results of the diagnosis. We can discern that all the categories emerging from the parents’ comments address the pain and difficulty posed by this challenge of raising a child with special needs. Overall, one may say that the parents are flooded with emotions, cognitively concerned, and active in coping with the diagnosis and what they think this means for them. 

That being so, this study combined categorical and qualitative data: the categorical analyses showed that most parents indeed achieved resolution with their child’s diagnosis. The categories included emotional, mental, and practical resolution. However, in the qualitative analysis, which delved into the topics preoccupying the parents according to the above categories, most parents mentioned guilt, shame, fear of stigma and concern for the child’s future, concealment, attempts to alter the diagnosis, and so forth. 

Thus, there seems to be a mismatch between the categorical findings indicating that the majority of parents had reached resolution and the qualitative findings showing characteristics that suggest non-resolution with their child’s diagnosis. This provides important innovation for understanding the process that such parents undergo following receipt of the diagnosis. The process is complex, involving ups and downs along the way, so that even after parents have reached resolution, their daily life still keeps them engaged with content seemingly linked to non-resolution. We may conclude from this that resolution indicates a parent’s ability to function in light of their child’s diagnosis, accept it, accept the child as is, and manage the child’s life in an optimal manner. At the same time, and apparently without contradiction, the parent undergoes complex experiences and must deal with fears and challenging emotions. These findings align with earlier studies which have clarified that the parents’ process of coping with raising a child with special needs is complex [3,4,23].

However, although this point has been specifically mentioned even within the RDI manual [22] and is evident when working with parents, a thorough literature review has found no studies discussing the continual strain and negative thoughts and feelings that accompany parents, even after they come to a resolution regarding their child’s diagnosis. 

Therefore, the current study is important on different levels; first, as mentioned, thanks to the combination of categorical and qualitative methodologies, unique findings emerged showing the complexity of the process parents undergo. Second, the insight that someone in a complex state is able to accept, process, and manage such a challenge while experiencing harsh content that seemingly contradicts that acceptance is of considerable importance. This insight is relevant to states of coping with difficulties in life in general and that of parents of a child with special needs in particular. Moreover, these findings might contribute to the work of counsellors in terms of their empathy toward the possible fluctuations in coping, even among parents who have already reached resolution. In addition, the counsellors might become more alert and better prepared for situations in which parents who have reached resolution experience moments of crisis. 

### Research Limitations and Future Directions

This study has several limitations. First, it should be noted that the differences found between the RDI categorization and the content analysis findings may stem from retrospective interviewing. Furthermore, the current sample is limited regarding representation. The participants were parents of children diagnosed with ASD and/or IDD. It would be interesting to examine whether these findings are replicated in families of children with other disabilities. In addition, this study focused on parents whose child was diagnosed in the previous two years. It may be insightful to examine parents’ reactions at different time intervals after the diagnosis. As on many occasions, our participants consisted mostly of mothers (40) and fewer fathers (20), fathers’ representation in such an inquiry should be stronger in order to obtain more gender-balanced findings. Finally, no single parents were recruited to our sample. Single parents are a unique and important population that is not represented in this study. Future quantitative research can shed light on the impact of variables such as birth order, child gender, etc.

To gain a more detailed and in-depth understanding of the parental dynamics regarding the resolution of their child’s diagnosis, we recommend conducting supplemental research examining the personal parental variables that might affect the parents’ degree of resolution with their child’s diagnosis, such as religious faith and their personal profiles. 

## 5. Conclusions

The aim of this study was to dive into the thoughts, feelings, and actions of parents of children with special needs surrounding their child’s diagnosis. Our findings point to inconsistencies within the parents’ reactions as a possible characteristic of the complex procedure the parents undergo. It is our sincere hope that the results of this study will be used to design instructional programs for education and health counsellors striving for better parent–counsellor partnerships. Based on the findings of the study, we propose the following recommendations for professional counsellors who accompany parents: First, understanding parental coping styles together with conducting empathic discourse adapted to the relevant coping style may make the dialogue more effective by enhancing the interaction between parents and counsellors. Second, understanding the complexity of the coping process, they should be aware that parents’ display of shame, guilt, or concealment does not mean that they are not in the process of achieving resolution regarding their child’s diagnosis. Conversely, when a person shows resolution, it does not necessarily mean that they are not overwhelmed by harsh content, including emotional breakdown, confusion, and thoughts about the diagnosis. We believe these are important insights for counsellors working with parents that might lead to greater empathy, inclusion, and understanding, as well as interventions that match parental behaviours, which often seem to be inconsistent and unstable. The understanding of these findings clarifies that parents of children with special needs can benefit from tailored support throughout their whole parenting journey. 

## Figures and Tables

**Table 1:** Frequencies and proportions of the RDI categories.

	Subcategory	Frequencies	Proportion (%)
**Resolved**	Emotional orientation	14	22.6
	Thinking orientation	14	22.6
	Active orientation	9	14.5
	Total	37	59.7
**Unresolved**	Emotionally overwhelmed	9	14.5
	Angry	2	3.23
	Depressed/passive	3	4.8
	Neutralizing	2	3.23
	Disorganized/confused	3	4.8
	Cognitive distortion	6	9.7
	Total	25	40.3

**Table 2:** Frequencies and proportions of the RDI categories by continuum.

Coping Style	Frequencies	Proportion (%)
Emotional	25	40.3
Cognitive	25	40.3
Active	12	19.4

## Data Availability

All data are available (in Hebrew—local language) by contacting the researchers.
